# Multimodal Emotion Recognition Based on Cascaded Multichannel and Hierarchical Fusion

**DOI:** 10.1155/2023/9645611

**Published:** 2023-01-05

**Authors:** Xia Liu, Zhijing Xu, Kan Huang

**Affiliations:** College of Information Engineering, Shanghai Maritime University, Shanghai 201306, China

## Abstract

Humans express their emotions in a variety of ways, which inspires research on multimodal fusion-based emotion recognition that utilizes different modalities to achieve information complementation. However, extracting deep emotional features from different modalities and fusing them remain a challenging task. It is essential to exploit the advantages of different extraction and fusion approaches to capture the emotional information contained within and across modalities. In this paper, we present a novel multimodal emotion recognition framework called multimodal emotion recognition based on cascaded multichannel and hierarchical fusion (CMC-HF), where visual, speech, and text signals are simultaneously utilized as multimodal inputs. First, three cascaded channels based on deep learning technology perform feature extraction for the three modalities separately to enhance deeper information extraction ability within each modality and improve recognition performance. Second, an improved hierarchical fusion module is introduced to promote intermodality interactions of three modalities and further improve recognition and classification accuracy. Finally, to validate the effectiveness of the designed CMC-HF model, some experiments are conducted to evaluate two benchmark datasets, IEMOCAP and CMU-MOSI. The results show that we achieved an almost 2%∼3.2% increase in accuracy of the four classes for the IEMOCAP dataset as well as an improvement of 0.9%∼2.5% in the average class accuracy for the CMU-MOSI dataset when compared to the existing state-of-the-art methods. The ablation experimental results indicate that the cascaded feature extraction method and the hierarchical fusion method make a significant contribution to multimodal emotion recognition, suggesting that the three modalities contain deeper information interactions of both intermodality and intramodality. Hence, the proposed model has better overall performance and achieves higher recognition efficiency and better robustness.

## 1. Introduction

Emotion recognition is one of the key components of human-computer interaction systems, which have a wide range of applications. At the same time, people are increasingly expecting interactive robots to have similar understanding capabilities and rich emotions as humans, thus putting forward higher requirements for human-computer interaction technology. However, because of the complex emotional characteristics of speakers, humans express emotions in a variety of ways, including language and facial expressions; therefore, it is not enough for existing service robots to mechanically rely on one modality for human-computer interaction, and a lack of understanding of semantic context and ignoring different pieces of emotional information contained in other different modes result in a less intelligent recognition process [1]. Emotion recognition remains a challenging task in human-computer interaction, as the speaker's emotional information is always reflected in different modalities. Comprehensively, considering multiple data modalities such as text, speech, and facial expression images can effectively improve the performance of emotion recognition.

The emotion recognition system usually consists of four consecutive modules: data processing, data encoders (feature extraction), feature fusion, and classification modules. The core issue of emotion recognition lies in how to adequately extract the information within a single modality and how to effectively fuse cross-modality information. In recent years, neural networks and various natural language processing techniques have shown good performance in the area of emotion recognition. This is attributed to the power of convolutional neural networks, attention mechanisms, and their various variants to model intramodality and intermodality emotion information.

Earlier mainstream works concentrated on unimodal emotion recognition, which focused on designing handcrafted features for emotional expressivity. Han et al. [2] extracted 238 low-level descriptor (LLD) speech description features using the openSMILE [3] tool and used connectionist temporal classification (CTC) to align these features with emotion labels. To develop automatic feature learning techniques, researchers are paying much attention to utilizing deep learning (DL) algorithms to obtain high-level features for speech emotion recognition. Han et al. [4] employed deep neural networks (DNNs) to extract deeper features from raw data and verified the effectiveness of DNNs in speech emotion recognition. However, this model failed to capture the long-distance interdependence information of the speech. Lee and Tashev [5] fully considered the long sequences of speech features and the uncertainty of emotion labels and proposed a method for emotion recognition based on recurrent neural networks (RNNs). Trigeorgis et al. [6] proposed a solution to speech emotion information extraction based on the convolutional neural network (CNN), which uses CNN combined with the long short-term memory (LSTM) network to identify local emotional information from the speech's contextual information. Neumann and Vu [7] designed CNN with multiview learning objective functions to compare the performance of different lengths of input signals, different types of acoustic features, and different types of emotional speech systems. Tashev et al. [8] constructed a system combining a low-level feature extractor of the Gaussian mixture model (GMM) with a high-level feature extractor based on DNNs to learn about the emotional features of speech. In traditional text emotion analysis research, extracted emotion features are based on the frequency of each word in the different text, which are obtained by statistics, such as word term frequency (TF) and word term frequency-inverse document frequency (TF-IDF), and then using logistic regression and the support vector machine (SVM) combined with other methods for emotion classification. Hutto and Gilbert [9] constructed a model based on a rule and compared it with 11 typical practice state benchmarks. In recent years, with the increasing availability of large amounts of training data, the use of deep learning to automatically extract features has gained popularity. Textual features have been mainly represented using global vectors (GloVe) [10] and Word2Vec [11] for word representation, which are based on global information. Kim [12] drew on their experience in image research and then used CNN's convolutional layers and downsampling layers to extract textual features and Word2Vec to transform textual data into the form of word vectors and perform emotion classification. With the development of artificial intelligence technology, current research on visual emotion analysis focuses on facial expression; in traditional visual emotion methods, such work mainly focuses on analyzing features, local features, and feature faces for face localization detection, after turning to handcrafted features, usually by calculating the offset of the eyes, eyebrows, mouth opening and closing degree, and other quantitative forms to represent expression features. The following are some of these methodologies based on traditional emotion methods. For example, the computer expression recognition toolbox (CERT) proposed by Littlewort and Wu [13] can automatically encode facial movements and facial expressions from the facial action coding system (FACS). Researchers integrate the three steps of face location, face detection and feature extraction into an end-to-end emotion recognition system. Because of this, Pushpa and Priya [14] used Deep Boltzman Machine (DBM) and CNN for emotion analysis. Compared with traditional methods, deep learning methods greatly improved the ability of emotion analysis. To enhance the performance of prediction models, the majority of the works turn to analyzing the pretrained model. Yu and Zhang [15] proposed a method that contains three face detection modules as well as multiple classification modules of deep CNNs, where each CNN model is pretrained on one dataset and then fine-tuned on another dataset in order to further extract its facial expression features. This model fully extracts the facial emotion feature after going through the pretraining and fine-tuning process, which results in better performance. Many researchers have extracted 3D features of expressions by CNN [16, 17], as well as combining RNNs for expression features of faces [18–20]. In addition, Jung et al. [21] proposed a DNN containing two models, one of which can extract feature emotions directly from original continuous face sequences and the other model extracts temporal geometric features from time-series face markers, and experiments proved that combining two models can give better results in face expression recognition. On the basis of neural network models such as CNN, RNN, and LSTM, more and more researchers have proposed network models such as the visual geometry group network (VGG-Net) [22] and ResNet [23] and constructed relevant emotion analysis models based on these models, and among them, the most representative network is the VGG network. VGG was proposed by Oxford's Visual Geometry Group in 2014. It won second place in the 2014 ImagNet large-scale visual recognition challenge. Compared with AlexNet, VGG uses a deeper network structure, which proves that increasing network depth can affect network performance to some extent. So far, VGG has not been used to extract image features; though there are many parameters, the network can effectively reduce the number of iterations required for convergence and greatly improve the computational speed, which has a better generalization ability.

Although the abovementioned deep learning algorithms perform well in extracting feature information from each modality, there are still many problems. Applying only a single type of network, e.g., CNN or LSTM, to speech and text information extraction cannot capture both long-distance information and local information and cannot accurately capture the expression changes of human faces in video modalities, and missing the contextual key emotional information and the key frame would result in some limitations in the feature extraction process. Besides, the parallel processing speed of such a modality is slow, resulting in greatly limited access to deep emotional information about each modality.

Existing emotion recognition methods mainly emphasize feature extraction of information within modalities, fail to effectively utilize complementary features between modalities, and ignore important sentiment information and differential sentiment features between different modalities. Therefore, how to establish a model that can effectively extract multimodal feature information and fully interact and fuse it is another challenge for emotion recognition.

To overcome the above two challenges, in this work, we propose a multimodal emotion recognition model based on cascaded multichannel and hierarchical fusion (CMC-HF). The main contributions of this paper can be summarized as follows:

1. To address the problem of poor emotional information extraction in the process of intramodality feature extraction, we employ three cascaded models: the cascaded sequential attention encoder (CSA encoder), emotional field cascade encoder (EFC encoder), and parallel-cascaded encoder (PC encoder), which are based on pretrained models with large-scale data combined with other deep learning networks to extract text, speech, and visual emotional features, respectively.
2. We propose hierarchical fusion to effectively learn about intermodal information interaction and fuse the important and contextual information of text, speech, and visual modalities for better recognition and classification.
3. We make an evaluation and comparison of robustness and generalizability of our model on two publicly available multimodal datasets and conduct a series of ablation studies to understand the effect of each major component on the architecture, and the proposed system model provides good performance and improves accuracy.

The remainder of the paper is organized as follows: In Section 2, we introduce the related work of emotion recognition.  Section 3 provides an overview of the proposed method and introduces the details of each module, respectively. Then, two emotional datasets are investigated to demonstrate the effectiveness and superiority of the proposed method in Section 4. Finally, the conclusions and future work are given in Section 5.

## 2. Related Work

As social media data in recent years are no longer limited to single modality, considering that these unimodal features based one network model was proved to be insufficient to precisely recognize the speaker's emotions. Some other modalities that can offer supplementary information are adopted to enhance recognition accuracy, so many researchers have turned to research on emotion recognition based on multimodal, such as speech and visual domains. Earlier studies [24, 25] have shown that fusing two modalities, speech and video, either in the feature layer [26] or in the decision layer [27], can help achieve higher recognition accuracy than that obtained from any single modality, and with the exploration of different modalities and different fusion methods, recognition accuracy is improving as well. Yoon et al. [28] used two RNN networks to encode information from speech and text sequences and directly connected the obtained sentiment feature sequences for emotion recognition, with a final recognition accuracy of 71.8%. Some studies have also attempted to pay attention to intramodal interaction to explore potential relationships. Yoon et al. [29] proposed multihop attention, which used attention mechanisms for successive interactions of text and speech modalities. Important features are learnt from one modality to another modality; the final accuracy rate was improved to 76.5%. Zadeh et al. [30] proposed a multinote block (MAB) to extract the interrelationships between expression, speech, and text modalities and store them in long and short time memory hybrid (LSTMMH). To enhance emotion recognition performance over unimodal or two bimodal methods, Poria et al. [31] exploited different methods to separately extract text, visual, and speech feature data and then performed decision layer fusion on the classified results. The authors in [32, 33] employed the same DL network for the three modalities for feature extraction. Majumder et al. [34] presented a novel feature fusion strategy that proceeds in a hierarchical fashion, first fusing two modalities and only then fusing all three modalities. Tsai et al. [35] used transformers to extract and fuse the features of the three modalities, which effectively solved the problems of unalignment of modal data and long-term dependencies between different modalities.

Also, recently, pretrained networks using transfer learning techniques have achieved good performance for extracting features [36], especially in the field of emotion recognition [37–40], and have advanced significantly. As the pretrained model can learn about global features from data, its parameters show better generalization effects.

In the process of multimodal emotion recognition, the pretrained network method is still insufficient when considering the emotional features of different dimensions, and further improvement is needed. So some researchers have suggested constructing some cascaded data encoders consisting of a combination of multiple models, which effectively take advantages of the benefits of each network. Different models focus on different dimensional features of the same input data, obtain richer sentiment features, and achieve better recognition results. Sun et al. [41] constructed a speech encoder by cascading CNN and LSTM to extract its deep semantic features and then fused it with text information in the feature layer, effectively capturing the sentiment information contained in speech, with a final accuracy improvement of 4%–5% compared to using only a single network as a data encoder. Lee et al. [42] used a BERT-based heterogeneous feature fusion approach to fuse multiple low-level features and high-level features of text, speech, and video for emotion recognition, and the final accuracy for the IEMOCAP dataset was up to 85.1%. Zadeh et al. [43] used the memory network to model speech, text, and video sequences, strictly aligned the three modalities in the time dimension, adopted LSTM for view-specific interactions, and proposed a dynamic memory-based attention network (DMAN) and a multiview-gated memory method for crossview interactions.

In the latest social networks, more and more multimodal emotion analysis is being conducted on videos through text, speech, and rich facial expressions [44]. However, multimodal research has a high standard for the size and diversity of corpora, and currently, available datasets are not enough to support broader research. Therefore, at present, the technology of extracting modalities from emotional features through pretrained networks combined with other deep learning technologies is still mainstream. Zhao et al. [45] proposed a novel multimodal transformer-based pretrained model, MEmoBERT, under self-supervised learning on a large-scale unlabelled movie dataset and further adopted a prompt-based learning method to adapt it to downstream tasks for multimodal emotion recognition, especially under low-resource conditions. Wei et al. [46] considered the computational and storage problem caused by a large number of long-length and high-resolution videos in the 5G and self-media era, and they transferred the success of transformer in vision into the audio modality and introduced the Rep VGG-based single-branch inference module for multimodal emotion recognition tasks; extensive experiments on the IEMOCAP and CMU-MOSEI datasets demonstrate the effectiveness of these methods, but for some similar emotional expressions like happy and neutral, the proposed model performs not so well in discriminating between these emotions. How to distinguish similar emotions and extract fine-grained information is also a problem that needs to be studied in future research. The work [47] explored the relationships between different modalities, adopted a bidirectional alignment network CNN-LSTM to obtain the aligned representation for different modalities, and showed empirically that the method has a discriminative power in emotion classification; inevitably, the alignment process would correspondingly increase the computing task.

In addition to the influence of an insufficient corpus, the multimodal emotion recognition study is also hindered by the mutual exclusion of different modalities, due to different modalities being involved in different domains. To further explore the context information and relationships between intermodalities, Liu et al. [48] designed a spatialtemporal graph convolutional network (SE-GCN) for video emotion recognition. By constructing two subnetworks, the sparse spatial graph and sparse temporal graph, an emotional relationship is obtained between any two emotion proposal regions and rich emotion cues. Then, SE-GCN is applied to obtain the reasoning features of the emotional relationship, due to GCN extending CNN to the graph structure. This method considers the relationship between different domains and explores the relationship between different modalities, which effectively improves the performance of multimodal emotion recognition. Inspired by the above work, GCN combined with other natural language processing (NLP) aimed to fuse frequency-domain features, time-domain features, and spatial features for comprehensive emotion analysis is constructed in our paper.

In short, although existing emotion recognition methods have made major breakthroughs, they have certain limitations. The data after being aligned are too costly and lead to a poor extraction process for extracting less and insufficient information feature with long-distance dependence. The feature data extracted by a single end-to-end network model are not comprehensive, rich enough, and deeper. In addition, the intermodal interactions of modalities are ignored, which would result in less attention being paid to emotional features. Therefore, in this paper, we consider that different modalities contain different emotional information, exploit the complementarity between intramodal and intermodal sequences, focus more on contributing features containing emotional information in the multimodal fusion stage, employ a hierarchical fusion approach for multimodal features, and finally enhance the accuracy of emotion recognition.

## 3. Materials and Methods

**Figure 1:**
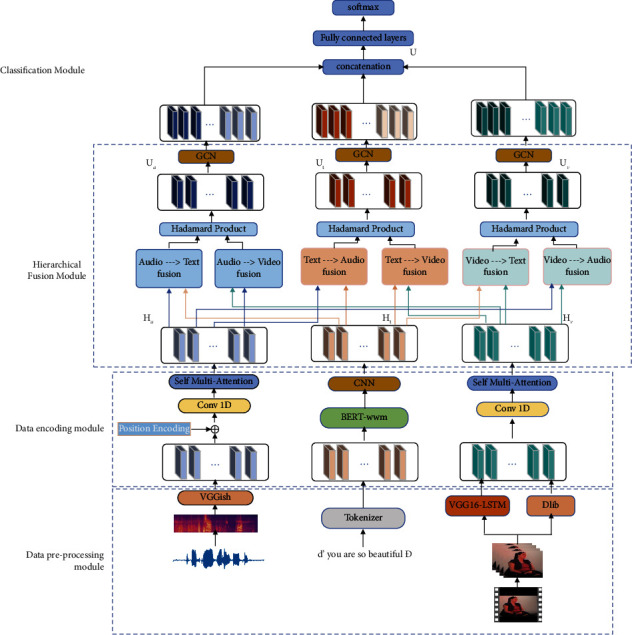
The architecture of the CMC-HF model proposed in this paper.

The pipeline of the proposed CMC-HF method in this paper is shown in Figure 1. There are four modules in our CMC-HF model: the data pretraining module, data coding module, data hierarchical fusion module, and emotion classification module. First, speech, text, and visual data are preprocessed. The cascaded encoder is used to extract the features of the three modalities' information separately to obtain their deep feature representations. Then, the collaborative attention mechanism is used to interact with the sequence information of any two modalities among the three modalities. After that, the Hadamard dot product is applied to extract the two-by-two interactive information belonging to the same central modal, and inter-bimodal information is input to the GCN for secondary fusion; the hierarchical fusion strategy that we proposed fully considers different and correlated emotion information between the three modalities. Finally, after intramodality and intermodality information interaction, the fused feature representation is concatenated and inputted into the two fully connected layers and the SoftMax classifier to achieve multimodal emotion recognition.

### 3.1. Data Preprocessing Module

#### 3.1.1. Text Data

We perform tokenization for text data to clean and segment text data and then split the text information into tokens, which can be used for BERT feature extraction. The operation is as follows:

  Step 1: We pretrain the BERT-wwm [49] model and import other corresponding models and classifiers.
  Step 2: For the original text data, we first split the sentence into several words, remove the symbols and text words that are without emotional information, and add four auxiliary tokens for the sentence (PAD for padding, CLS for sentence beginning, SEP for sentence spacing, and MASK for obscuring words).
  Step 3: We embed words and convert each sentence into a fixed-length sequence. After the embedding operation, each word in the sentence is represented as a word vector with a length of 768.

#### 3.1.2. Speech Data

For speech data, we extract the spectrogram from them and then input them into a pretrained deep network VGGish [50] to extract shallow speech features, which can obtain an embedded vector containing semantic information. VGGish is a model trained on AudioSet, a large sound dataset published by Google, which can extract rich semantic information from speech data. The speech preprocessing process is as follows:

  Step 1: We resample the speech file in 16 kHz mono, use the 25 ms Hamming window, and perform a short Fourier transform of the speech with 10 ms frame shift to obtain a spectrogram.
  Step 2: We calculate the log spectrogram with a size of 96 × 64; after inputting the spectrogram into the VGGish network, we extract a 128-dimensional embedding vector containing rich semantic information about the last fully connected layer as the input to the next network.

#### 3.1.3. Visual Data

For visual data, we decided to analyze facial expressions in the video for visual emotional analysis. Since human express emotion is a process and the facial expression change is actually slow and continuous, we grab the effective frames in the video in a specific way. Specifically, we sample continuous video frames at certain intervals and analyze these video frames as visual emotional features. On the one hand, capturing a subset of the video frames can reduce the requirements of computer resources; on the other hand, it can prevent some additional noise data being extracted from unnecessary redundant features, which may affect the accuracy of emotional recognition. The specific video preprocessing methods are as follows:

  Step 1: We read all the video clips in the dataset and get the frame rate of each video; after calculating the average frame rate of the dataset, we trim the length of each video according to the average frame rate, intercept the video that exceeds the average frame rate at the end, and pad zero frames for the video that is less than the average frame rate.
  Step 2: We follow the method that one frame is sampled every two frames to sample the processed video, and then, each video retains 32 frames for feature extraction. We use the OpenCV library to read the video frame data, and finally, a single video sample is processed into a data form with dimensions (224, 224, 3, 32).

### 3.2. Data Encoding Module

#### 3.2.1. Speech Encoder

After preprocessing the speech data, we add position encoding to encode the temporal position of words for characterization and apply the one-dimensional temporal convolutional network to transform the speech feature dimension to obtain a sequence dimension *d*, which is consistent with text features and can be expressed as follows:

(1) $$\begin{array}{c} {X^{\prime }}^{a}=Conv1D\left(X^{a},k^{a}\right)\in R^{T^{a}\times d_{a}}\text{,} \end{array}$$

where *k*^*a*^ represents the convolutional kernel size of the convolutional network, *X*^*a*^={*x*_1_^*a*^, *x*_2_^*a*^, ⋯, *x*_*T*^*a*^_^*a*^} ∈ *R*^*T*^*a*^×*d*_*a*_^ is the input speech feature, and *T*^*a*^ and *d*_*a*_ are the speech sequence length and feature dimension, respectively.

**Figure 2:**
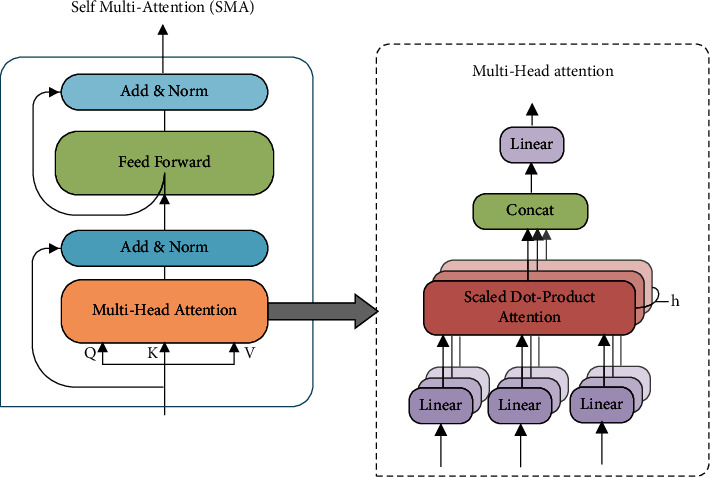
Transformer encoder.

In the process of speech sequences feature extraction, traditional recurrent neural networks such as LSTM have a poor parallel computing capability because of the limitation of the sequence length, which cannot capture the relationship between long-distance words in a sentence and lack the understanding of semantic information. Therefore, in this paper, we use transformer encoders to encode extracted speech features and parallelize the word sequences in sentences. Then, we combine them with the VGGish network to form CSA-encoder to extract deep semantic information from the text. On the one hand, it can effectively solve the problem of insufficient semantic extraction for distance limitation, fully understand the contextual relationship between words, and extract rich semantic information; on the other hand, the multihead attention mechanism in the transformer encoder pays different attention to features, which can assign different weight sizes to words containing different degrees of emotional information. Different heads pay attention to different emotional information, and after concatenation, the model can obtain rich emotional information features in speech sequences. The structure of transformer encoder self-multi attention (SMA) is shown in Figure 2, which mainly consists of the multihead attention mechanism and fully connected neural networks.

The multihead attention mechanism computes input sentiment sequences in parallel compared with the traditional self-attention mechanism that only acquires attention information from a single level. The multihead attention mechanism has multiple heads, and each of which pays different attention to the input speech emotion information and can generate different self-attention distributions, thus obtaining more comprehensive emotion information. The attention value of the *i*th head is calculated and is executed in scaled dot-product calculation as follows:

(2) $$\begin{array}{c} head_{i}=softmax\left(\frac{Q_{a}{K_{a}}^{T}}{\sqrt{d_{k}}}\right)V_{a}\text{,} \end{array}$$

where *Q*_*a*_=*X*^′*a*^ *W*_*i*_^*Q*^, *K*_*a*_=*X*^′*a*^ *W*_*i*_^*K*^, and *V*_*a*_=*X*^′*a*^ *W*_*i*_^*V*^ are three vectors generated by the linear variation of the speech sequence, corresponding to the query, key, and value of the feature sequence of the modality *a* and *W*_*i*_^*Q*^ ∈ *R*^*d*×*d*_*k*_^, *W*_*i*_^*K*^ ∈ *R*^*d*×*d*_*k*_^, and *W*_*i*_^*V*^ ∈ *R*^*d*×*d*_*k*_^ are the corresponding projection matrices.

Then, the attention values obtained from *m*(*d*_*k*_=*d*/*m*) heads are concatenated together and multiplied by the corresponding weight matrix *W*^o^ ∈ *R*^*d*×*d*^ to obtain intramodal attention ${\overset{\wedge }{X}}^{a}$:

(3) $$\begin{array}{c} {\overset{\wedge }{X}}^{a}=Concat\left(head_{i},\cdots ,head_{m}\right)W^{o}\text{.} \end{array}$$

Finally, we input ${\overset{\wedge }{X}}^{a}$ to the fully connected feedforward neural network (FNN), and after two times of linear variation using ReLU, we can obtain the final deep feature representation of speech *H*_*a*_={*h*_1_^*a*^, *h*_2_^*a*^, ⋯, *h*_*T*^*a*^_^*a*^} ∈ *R*^*T*^*a*^×*d*^:

(4) $$\begin{array}{c} H_{a}=FFN\left({\overset{\wedge }{X}}^{a}\right)=\max\;\left(0,{\overset{\wedge }{X}}^{a}W_{1}+b_{1}\right)W_{2}+b_{2}\text{.} \end{array}$$

Note that *W*_1_, *W*_2_ and  *b*_1_, *b*_2_ are the trainable weight matrices and deviations of the fully connected layers, respectively.

#### 3.2.2. Text Encoder

We choose BERT whole word masking (BERT-wwm) as a feature extraction network for text. It is an upgraded version of BERT released by Google in 2019, which mainly changes the sample generation strategy of the original pretraining phase. BERT, a transformer-based model, has strong language representation and feature extraction ability and has shown performance improvement by fine-tuning pretrained weights for a specific downstream task, which now achieves state of the art in eleven NLP benchmark tasks. BERT introduces two core tasks in the pretraining stage, one is masked LM (language model training with mask) and the other is next sentence prediction. In the mask operation, the original word splitting method of the BERT pretraining model [51] is based on WordPiece, which splits a complete word into several subwords blindly, and mask operations are randomly performed on these separated subwords to generate training samples. However, this splitting method is still not suitable for English expressions because a word contains various root and affix variants. Some adjectives or adverbs containing emotional information have more forms. For example, some adverbs denote the highest degree like “completely” and “deeply,” some adverbs denote the middle degree like “somewhat” and “merely,” and some adverbs denote the least degree like “hardly” and “nearly.” In the traditional way of word splitting, the adverb “somewhat” is divided into the subwords “some” and “what,” and then randomly masked during the training process. It is obvious that this training method does not allow the model to learn the semantic information in the text, so we use the BERT-wwm model in this paper. When the subwords in a word that contains emotional information are masked, the other subwords belonging to the same word will also be masked accordingly, which can improve the adaptability of the pretraining model in extracting the key emotional information and better solve the problems of semantic ambiguity and sparsity for text emotion key features.

In order to make the BERT pretraining model better to extract emotional key features for subsequent sentiment recognition, in addition to the pretrained BERT model given by the official, we also use the IEMOCAP and CMU-MOSI datasets to train the model again. The generated sample is shown in Table 1.

From Table 1, we can find that the word vector obtained by the BERT-wwm pretraining model effectively reduces the loss of emotional information and captures more emotional features.

**Figure 3:**
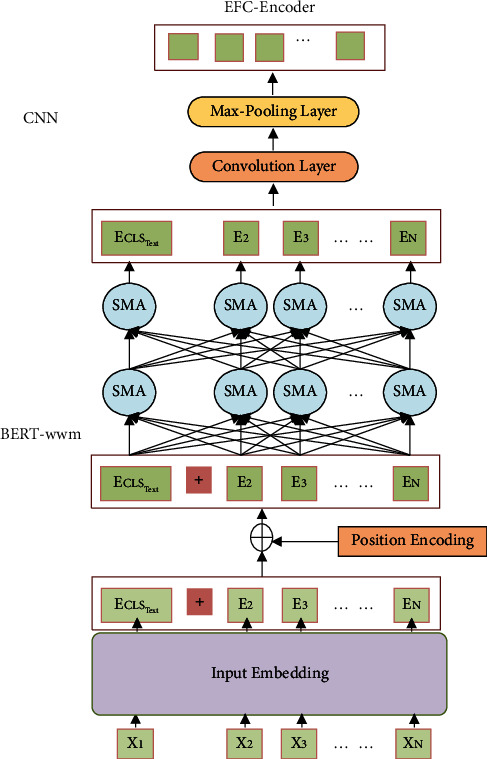
EFC encoder framework.

However, transformer blocks in BERT-wwm mainly emphasize the model's ability of long-distance dependence, which pays attention to the global features of word vectors, not local information features; therefore, on the basis of the abovementioned BERT-wwm pretraining model, we introduce the CNN to further perfect the inadequacies of a single-text extraction network, as the sliding window mechanism in CNN can effectively focus on the local information features of word vectors. The specific network structure is shown in Figure 3, and a convolutional layer and a maximum pooling layer are connected behind the BERT-wwm structure to form the EFC encoder. It can improve the ability to extraxt contextual information around a word and reduce the loss of emotional information. On the other hand, the global and local information can be considered, effectively improving final recognition accuracy.

The specific calculation process of extracting text features from the EFC-encoder network model is as follows: The original text sequence *utt*_*i*,*s*_ = (*s*_1_, *s*_2_,…, *s*_*n*_) is tokenized to *X*^*t*^ = {*x*_1_^*t*^, *x*_2_^*t*^,…, *x*_*T*^*a*^_^*t*^}, *t* ∈ *R*^*T*^*t*^×*d*_*t*_^. Then, as the BERT-wwm pretraining model's input, it can obtain the word vector with rich global information and capture the long-distance information. After that, we exploit the CNN to extract the local text information which captures emotional features missed by the BERT-wwm network and finally obtains textual features containing rich emotional information representation *H*_*t*_ = {*h*_1_^*t*^, *h*_2_^*t*^,…, *h*_*T*^*a*^_^*t*^} ∈ *R*^*T*^*a*^×*d*^:

(5) $$\begin{array}{c} {\overset{\wedge }{X}}_{t}=BERT\left(f_{BERT-wwm,}\left(utt_{i,s}\right),h\right)\text{,}\\ H_{t}=ReLU\left(\overset{\wedge }{X}W^{f}+b^{f}\right)\text{,} \end{array}$$

where *h* = 1,2,…, 24; *i* = 1,2,…, *n*, *f*_BERT−wwm_(*utt*_*i*,*s*_) indicates making tokenization to the input's sequence, and *h* is the number of implicit layers of the transformer encoder in BERT. We choose the model that contains 12 transformer blocks, 16 attention heads, and 340 million parameters and select the output of the last four transformer encoder layers' hidden layer of the model as the input for the next step. We get the word's length as 3072; the CNN convolutional kernel size is [3 4 5], and the number of convolutional kernels is 128. We use the ReLU as the activation function.

#### 3.2.3. Visual Encoder

This paper uses two parallel channels to extract the features of the preprocessed visual data: one of which adopts the cascade model VGG16-LSTM to extract deep learning features of the visual data in the entire image; another uses the VGG16 network pretrained on ImageNet to extract the each frame's representation information from the image, as it has been pretrained by large-scale data and introduced so much sufficient prior knowledge, which obtains more abundant emotion representation information for the model and provides more robustness for the sentiment analysis results.

In addition, the LSTM network effectively increases the time dimension information for visual emotional features, adds more timing information for expression emotional features, and makes up for shortcomings of insufficient feature extraction of a single image after video framing. The VGG16 network extracts 4096 dimensions of facial expression features as the LSTM's input, and we can obtain the final facial emotional features *X*_VGG16−LSTM_^*V*^={*x*_VGG16−LSTM_^*v*,1^, *x*_VGG16−LSTM_^*v*,2^,…, *x*_VGG16−LSTM_^*v*,*T*^*V*^^ ∈ *R*^4096^}.

After performing the experiment and data analysis of the aforementioned operations, we find that the aforementioned process is the extraction of emotional features from the whole image in a certain frame, not the people's faces. However, when performing visual emotion analysis, the emotion expressed by the face should be emphasized, and because the image contains a lot of irrelevant backgrounds, objects, and other noises, it cannot provide effective information, which will directly affect the effect of emotion analysis.

Therefore, in order to further accurately analyze the changes in facial emotion, we introduce corresponding auxiliary features to eliminate the influence of these noise data.

The Dlib library can detect the facial information in each frame of the image, which only extracts the facial expression feature and ignores background information. After collecting the 68 key point emotional information characteristics of the face and obtaining detailed coordinate information for each key point, including the nose, eyebrows, the outline of the face, eyes, and other information, and then using these key point coordinates to calculate the coordinates of a centre point, we obtain the distance information between the centre point and each key point; finally, we combine the obtained distance information as the emotional feature representation. The length of this feature vector is 68, noted as *X*_Dlib_^*V*^={*x*_Dlib_^*v*,1^, *x*_Dlib_^*v*,2^*x*,…, *x*_Dlib_^*v*,*T*^*V*^^ ∈ *R*^68^}.

Then, the two channels VGG16-LSTM and Dlib library jointly constituted the PC encoder to extract visual features in parallel, and the final expression emotion feature can be expressed as *X*^*V*^=(*X*_VGG16−LSTM_^*V*^, *X*_Dlib_^*V*^) ∈ *R*^*T*^*V*^×4164^.

Considering that facial emotional change is a dynamic process, there is a strong temporal correlation between the sampled frames in the same video, after inputting *X*^*V*^ to a 1D CNN, it can convert the visual modality's dimension into the same one as the text modality. In the self-attention mechanism module, we can pay attention to the association between the current visual frame and others. According to the number of sampled video frames, the facial emotion feature vector is converted into N groups, and the size of each set of feature vector is $\overset{-}{v}=v/N$, where *v* represents the dimension of the facial emotion feature vector and N is the number of sampled video frame frames. The facial attention value *H*_*v*_ can be obtained as follows:

(6) $$\begin{array}{c} {X^{\prime }}^{V}=Conv1D\left(X^{V},k^{V}\right)\in R^{T^{V}\times d}\text{,}\\ \alpha_{t}^{v}=\frac{\exp\;\left(W^{v}.X_{t}^{v}\right)}{\sum_{t=1}^{N}\exp\;\left(W^{v}.X_{t}^{v}\right)}H_{v}=\overset{N}{\underset{t=1}{\sum }}\alpha_{t}^{v}X_{v}^{t}\text{,} \end{array}$$

where *k*^*V*^ represents the convolutional kernel size of the 1D CNN, *T*^*V*^ and *d*_*V*_ are the length and feature dimension of the video data sequence, respectively, *α*_*t*_^*v*^ is the weight coefficient of the eigenvector of group *t*, *X*_*v*_^*t*^ represents the eigenvector of group *t*, and *W*^*v*^ is the trainable linear transformation parameter vector.

### 3.3. Hierarchical Fusion Module

At present, much work in the multimodal fusion stage only simply connects the feature sequences of different modalities, does not make full use of the important emotional and different characteristics between different modalities, and ignores the interaction of different modalities. In addition, traditional fusion methods are mostly based on aligned multimodal data, which fused according to time series, which would cost too much resources. To overcome this issue, we employ a novel hierarchical fusion strategy to explicitly model the intermodal interactions of the three modalities. Specifically, hierarchical fusion first utilizes collaborative attention to guide one modality to attend to the other modality and update features accordingly, and six fusion modules are obtained, as illustrated in Figure 1; the six fusion blocks can be grouped into three pairs considering their core modality (modality of the *Q* vector in the collaborative attention computation). As the next step, to extract the essential information that stems from one modality, we take the Hadamard product between fusion module pairs of the same core modality. Finally, we employ the GCN to further fuse the information of the three modalities in a cross-domain way. In the following part, we introduce the hierarchical fusion strategy in detail.

#### 3.3.1. Collaborative Attention

First, we consider the important emotional characteristic information for any two modalities. In the first fusion stage, we employ the collaborative attention mechanism to attend to cross-modal interactions at different time steps, propagate information between the two modalities, and capture long-distance information, which aims to let the model learn to pay more attention to more informative modalities in the fusion process, and the six fusion blocks can be expressed as COAM_*i*⟶*j*_, where *i*, *j* ∈ {*l*, *a*, *v*}.

**Figure 4:**
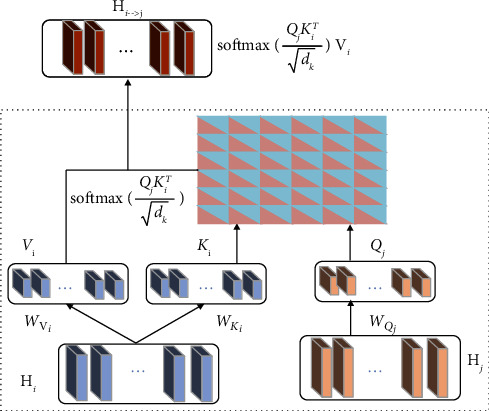
The calculation process of COAM_*i*⟶*j*_(*H*_*i*_, *H*_*j*_).

The collaborative attention mechanism module is mainly composed of *N* transformer encoder blocks, which include *N*-stacked cross-attention layers, feedforward layers, and a position embedding layer. Single-head cross attention can be calculated by (7), which is illustrated in Figure 4.

(7) $$\begin{array}{c} COAM_{i⟶j}\left(H_{i},H_{j}\right)=softmax\left(\frac{Q_{j}K_{i}^{T}}{\sqrt{d_{k}}}\right)V_{i}\text{.} \end{array}$$

COAM_*i*⟶*j*_(*H*_*i*_, *H*_*j*_) represents the modality *i* that can receive important emotional information from core modality *j*, where query, key, and value vectors are *Q*_*j*_=*H*_*j*_*W*_*Q*_*j*__, *K*_*i*_=*H*_*i*_*W*_*K*_*i*__, and *V*_*i*_=*H*_*i*_*W*_*V*_*i*__ and *W*_*Q*_*j*__, *W*_*V*_*i*__, *W*_*K*_*i*__ ∈ *R*^*d*_mode l_×*d*_*k*_^ are the corresponding weight matrices.

As the collaborative attention mechanism module based on *N* transformer encoder blocks adopts the *N*-stacked cross-attention layers, there exists a multihead version of COAM_*i*⟶*j*_(*H*_*i*_, *H*_*j*_), and the process can be mathematically described as follows:

(8) $$\begin{array}{c} H_{i⟶j}^{\left[0\right]}=H_{j}^{\left[0\right]}\text{,}\\ {\overset{\wedge }{H}}_{t⟶a}^{\left[i\right]}=COAM_{i⟶j}^{\left[i\right],mult}\left(\text{LN}\left(H_{i⟶j}^{\left[i-1\right]},\text{LN}\left(H_{i⟶j}^{\left[0\right]}\right)\right)+\text{LN}\left(H_{i⟶j}^{\left[i-1\right]}\right)\right)\text{,}\\ H_{i⟶j}^{\left[i\right]}={f_{\theta }}_{i⟶j}^{\left[i\right]}\left(\text{LN}\right)\left({\overset{\wedge }{H}}_{i⟶j}^{\left[i\right]}\right)+\text{LN}\left({\overset{\wedge }{H}}_{i⟶j}^{\left[i\right]}\right)\text{,} \end{array}$$

where *f*_*θ*_ is the position feedforward network with the parameter *θ*, COAM_*i*⟶*j*_^[*i*],mult^ is the multihead attention of the *i*th layer, and LN is layer normalization. *H*_*i*⟶*j*_^[*i*]^ represents the output of the *i*th transformer encoder layer, where *i*={1,2,…*N*}; after passing through the collaborative attention mechanism module, we obtain a two-by-two fusion representation *H*_*i*⟶*j*_^[*N*]^ with the central modality *j* at the last layer of *N* layers.

According to the above process, we can also obtain a bimodal fusion representation *H*_*j*⟶*i*_^[*N*]^, which represents the propagated information from modality *j* to core modality *i*.

After the collaborative attention mechanism modules, we can obtain six two-by-two fusion modality interaction pairs, respectively, that is {*H*_*a*⟶*v*_^[*N*]^, *H*_*a*⟶*l*_^[*N*]^}, {*H*_*v*⟶*a*_^[*N*]^, *H*_*v*⟶*l*_^[*N*]^}, {*H*_*l*⟶*a*_^[*N*]^, *H*_*l*⟶*v*_^[*N*]^}. We can get three resultant vectors *U*_*a*_, *U*_*t*_, and *U*_*v*_, after computing the Hadamard product between the first fusion stage outputs that belong to the same core modality.

(9) $$\begin{array}{c} U_{a}=\left[U_{a⟶v}⊙U_{a⟶t}\right]\text{,}\\ U_{t}=\left[U_{t⟶v}⊙U_{t⟶a}\right]\text{,}\\ U_{v}=\left[U_{v⟶t}⊙U_{v⟶a}\right]\text{.} \end{array}$$

#### 3.3.2. Graph Convolutional Network

GCN is usually used to obtain a graph representation of its graph structure, which is done in a graph embedding way. However, with the growing popularity and development of GCN, it is also applied to the study of multimodal emotion analysis to obtain contextual information. Specifically, by constructing the graph structure relationship between adjacent samples, the GCN further captures different features contained between different modalities and learns about the fused multimodal contextual information. Moreover, the GCN can effectively combine frequency-domain features, time-domain features, and spatial features for comprehensive analysis, which has a significant role in multimodal emotion recognition.

First, we need to establish the structure relationship between adjacent sample graphs. In this paper, we divide a complete video into some short video samples, and each short video sample is regarded as an adjacent sample, as there is a sequential relationship in time series between the adjacent samples, which indicates that the currently expressed emotion is closely related to the previous and subsequent emotion, and this relationship can be mapped as a graph structure in the GCN. According to the above analysis, we use the bimodal emotion features belonging to the same core modality *U*_*a*_, *U*_*t*_, *U*_*v*_, and ∈*R*^*M*×*f*^ as the node set of the graph structure. *M* is the number of adjacent samples and *f* is the dimension of the bimodal emotion features. Then, the adjacency matrix *A*, which is used to define the edge set information *U*_*e*_ between adjacent samples, can be constructed by the following similarity formula:

(10) $$\begin{array}{c} sim=\frac{v_{i}v_{j}}{\left\|v_{i}\right\|\left\|v_{j}\right\|}\text{,} \end{array}$$

where *v*_*i*_ and *v*_*j*_ represent the multimodal emotion feature vectors of the ith and jth adjacent samples, sim represents the cosine similarity, and ‖.‖ represents a module operation; when sim ≥ 0.8, the value of *a*_*i*,*j*_ is 1, which is the element of the adjacency matrix *A*. When sim < 0.8, *a*_*i*,*j*_ is 0.

After constructing the adjacent sample graph structure, it is necessary to build a reasonable model to achieve emotional fusion, input the graph structure to the GCN, and perform a series of graph convolution operations. The convolution operation on any node or graph structure is completed by aggregating the edge set information of its adjacent nodes, which is shown in the following equation:

(11) $$\begin{array}{c} H^{l+1}=\sigma \left(D^{1/2}\left(D-A\right)D^{1/2}H^{l}W^{l}\right)\text{,} \end{array}$$

where the degree matrix is $D=\left(\begin{array}{ccccc} \underset{j}{\sum }a_{1,j} & \ldots & 0 & \cdots & 0\\ \vdots & \ddots & \vdots & \vdots & \vdots \\ 0 & \cdots & \underset{j}{\sum }a_{i,j} & \cdots & 0\\ \vdots & \vdots & \vdots & \ddots & \vdots \\ 0 & \cdots & 0 & \cdots & \underset{j}{\sum }a_{N,j} \end{array}\right)$, *a*_*i*,*j*_ represents the elements of column *j* of row *i* in the adjacency matrix *A*, *H*^*l*^ represents the *l*th convolutional layer output, and *H*^0^=*U*_*i*_, *i* ∈ {*l*., *a*, *v*}*U*_*i*_ is the bimodal emotion feature vector after the Hadamard dot product, *W*^*l*^ is a trainable linear change parameter, and *l* represents the number of layers of graph convolution. In the experimental process, the value of *l* is 0 or 1, and *σ*(.) represents the sigmoid nonlinear activation function. By overlaying the graph, convolutional layers enable the network to learn about context dependencies between adjacent samples.

Finally, three pieces of enhanced modal information are concatenated together to obtain a feature vector representation for emotion classification, which can be represented as follows:

(12) $$\begin{array}{c} U=\left[GCN\left(U_{t}\right),GCN\left(U_{a}\right),GCN\left(U_{v}\right)\right]\text{.} \end{array}$$

### 3.4. Classification Module

To perform final classification, we use two fully connected layers and a SoftMax classifier to predict the underlying emotion. The ReLU function is used as an activation function, which can effectively overcome disappearance of the training gradient and avoid gradient explosion. The predicted scores of each emotional label are calculated through the SoftMax layer. The cross-entropy loss is used to optimize the model. The above process is summarized as follows:

(13) $$\begin{array}{c} \overset{\wedge }{y}=softmax\left(f_{\theta }\left(U\right)\right)\text{,}\\ softmax\left(x_{i}\right)=\frac{e^{x_{i}}}{\sum_{i=1}^{c}e^{x_{i}}}\\ l=-\overset{C}{\underset{i}{\sum }}y_{i}\log\left({\overset{\wedge }{y}}_{i}\right)\text{,} \end{array}$$

where *e*^*x*_*i*_^*q* represents the output value of the *it*h node in the classification model, *C* is the number of emotion classes, and the value of *C* varies according to different datasets, y_**i**_ represents the real value, ${\overset{\wedge }{y}}_{i}$ represents the predicted value of the model, and *f*_*θ*_ represents a fully connected layer network with a parameter *θ*.

## 4. Results and Discussion

### 4.1. Datasets

In this paper, the proposed models are evaluated on the IEMOCAP and CMU-MOSI datasets, and dataset preprocessing operations are illustrated as follows.

#### 4.1.1. IEMOCAP

The IEMOCAP dataset contains two-by-two dialogues between 10 actors, with a total of 5 sessions, each of which is completed by 1 man and 1 woman. Sessions are utterances with a corresponding emotional label. There are usually 7 emotion classes of “happiness,” “excitement,” “frustration,” “sadness,” “neutral,” “anger,” and “others.” However, in the process of speech data preprocessing, we found that the spectrogram of “excitement” was similar to that of “happiness,” and the expression ways of these two emotions were similar; besides, the class “happiness” had a small sample number, so we combined the “excitement” and “happiness” samples, followed by “happiness.” The spectrum of “frustration” was similar to that of “sadness,” and the two were merged in the same way, followed by “sadness,” and did not consider the “others” class. After operating on unbalanced samples, we finally chose four emotion classes, namely, “happiness,” “anger,” “neutral,” and “sadness” as the IEMOCAP dataset's experimental emotion classes.

Then, we considered that there were a total of 5531 utterances of these four classes in five dialogues; therefore, we took an utterance as a unit, divided a video into multiple short videos, and used these short videos as samples. Similarly, we obtained the same number of text and speech samples and then used 4290 utterances (77.7%) of 8 actors in 4 sessions as the training set for the experiment. 1241 utterances (22.3%) of 2 actors in 5 sessions are used as the testing set. The details of the IEMOCAP dataset assignment are shown in Table 2.

#### 4.1.2. CMU-MOSI

The CMU-MOSI dataset is a multimodal emotion analysis dataset published in 2016 and contains 2199 video clips from single-lens commentary recordings on YouTube, as well as text from each video recorder's content. Unlike the common discrete emotion labels mentioned above, videos are segmented into utterances where each utterance is annotated with scores between −3 (strongly negative) and +3 (strongly positive) by five annotators. We take the average of these five annotations as the sentiment polarity and consider only two (positive and negative). We randomly select 1284 utterances for the dataset as the training set, including 1447 utterances, and select the remaining 915 utterances as the testing set. We also take an utterance as a sample and obtain these divided short videos as samples.

### 4.2. Experimental Details and Evaluation Metrics

The experiments are based on the Python framework launched by Facebook. The experimental platform used in this article is shown in Table 3.

In the speech and visual data encoder module, we set multihead attention heads to 8 and the size of attention heads to 64. The dropout of the model is 0.3, which can prevent overfitting, and the batch size of the model is set to 32. When training is completed up to 100 times, the accuracy of the model no longer increases, and it will stop in advance and save the training results. The learning rate is initialized to 0.00001, and when the training effect of the model no longer rises, the learning rate automatically decreases. The Adam algorithm, as an optimizer, is used to find optimal parameters.

In the process of selecting other important parameters, we unify the text sequence length to 20, for sequences with a length greater than 20, truncate the part with a length greater than 20. For sequences with a length less than 20, we select the pad zero operation. In addition, in the collaborative attention mechanism module, considering that the amount of multihead attention and the number of attention layers have a greater impact on the information interaction process; according to [32], too many heads and layers are easy to cause information redundancy. On the contrary, the lower number of heads and layers resulting in long-distance information between the two modalities has insufficient interaction, and long sequence information is not fully captured, resulting in the omission of valid information, so we choose the number of layers and heads of multihead attention in the collaborative attention mechanism module as five. We used the accuracy (Acc), *F*1 value (*F*1 score), mean average error (MAE), and correlation coefficient (Corr) as the main evaluation metrics.

### 4.3. Comparison with State-of-the-Art Approaches

To comprehensively evaluate our proposed CMC-HF approach, the following baselines and state-of-the-art approaches are utilized for comparison. The details of the baselines are shown in Table 4, and the experimental results are shown in Tables 5 and 6.

From Tables 5 and 6, it can be seen that the performance of the CMC-HF model proposed in this paper outperforms that of other state-of-the-art models for both benchmarks. Specifically, for the IEMOCAP dataset, it can be seen that the CMC-HF model has higher recognition accuracy than other models for the four emotion classes. As the MFN, MCTN, RAVEN, and MulT models mainly use a single traditional network (LSTM or CNN) to extract modality features, such networks fail to capture long-distance and local information at the same time in the speech and text modality and can easily miss the key frame information in the video. SSE-FT performs best in the existing SOTA models because it uses the same pretrained network method and the hierarchical fusion way (fuse different modality features in a phased form), which shows the hierarchical fusion way and achieves a better performance compared with the SSE-FT model. Neutral has the largest increase in recognition accuracy with an increase of 3%, and anger has a relatively small increase in recognition accuracy with an increase of 1.9%. Such excellent results confirm the superiority of the CMC-HF structure.  Table 6 shows the performance of the CMC-HF model for the CMU-MOSI dataset, where Acc (2 class h), *F*1 score, MAE, and Corr performed better than the above baseline model, and the value of Acc (7 class h) is little lower than that of the most advanced model.

**Figure 5:**
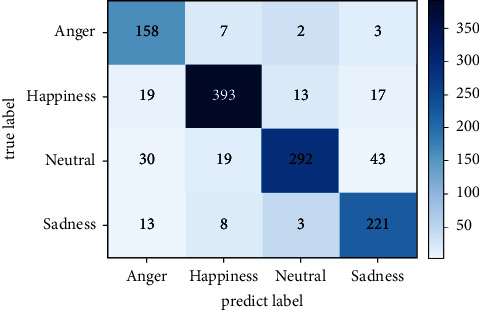
A confusion matrix diagram of four emotion classes for the IEMOCAP dataset.

In addition, the confusion matrix obtained by the IEMOCAP dataset is shown in Figure 5. The abscissa of the confusion matrix plot represents four class emotion labels predicted by the model. The darker the colour of the square on the diagonal, the higher the recognition accuracy of the corresponding category of the square. All emotion categories have been identified with a high level of per class accuracy, as indicated by the diagonal elements of matrices. In this case, because the text and speech modalities are more pronounced in the emotional performance of anger and sadness, the overall accuracy rate is higher than that of happy and neutral, and it is due to the fact that happy contains a wider range of text emotion expressions, while the neutral's emotional state is more ambiguous, resulting in the two being easily confused with other emotional classes, and prediction accuracy is relatively lower.

**Figure 6:**
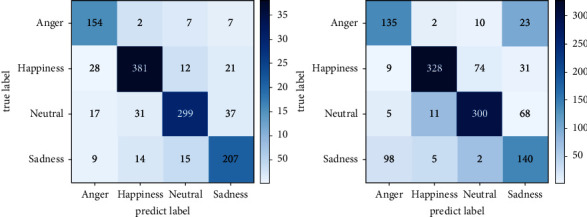
A confusion matrix of the SSE-FT and HFusion model for the IEMOCAP dataset.

We report confusion matrix comparison for the SSE-FT and HFusion models' performance on the more challenging IEMOCAP dataset. From Figure 6, we can infer that the neutral performance of the HFusion model is lower than that of the SSE-FT model, whereas performances of the other three classes are higher. Compared with the result of Figure 5, our proposed model achieves an excellent and competitive performance, which verifies the combination of modality data encoders and ensures that the learnt representations are compact, rich, and complementary, thus making the architecture optimal and robust towards the recognition task for multimodal data.

A potential limitation of our model may be its trainable weight and parameters. However, the number of parameters in our model is 132,491,017, which is 25% of the SSE-FT model and 60% of the HFusion model, which have reduced compared with that of these two cascade models. To explore the time complexity of CMC-HF, we compute FLOPs for these three cascade models in the testing process; the result turns out that our model needs 261,322,124 FLOPs in testing, whereas the number of FLOPs in HFusion and SSE-FT is 253,961,225 and 591,236,844, respectively. Undoubtedly, the inference time is a key factor for the emotion recognition task. For this parameter, we report an average of 63.2 ms through the whole process, whereas the HFusion and SSE-FT models yield a total of 98.4 ms and 132.9 ms, leading to an increased inference time. Considering the low inference time, the proposed CMC-HF model has better performance.

The reason for fewer trainable parameters, the moderate number of FLOPs, and the lower inference time is that despite having multiple components in our architecture, our model entails low dimensions for unimodal and multimodal representations. For unimodal representations, we apply conv 1D-unified feature dimension for speech and vision, respectively, and the CNN model is introduced to obtain the low dimensions for unimodal representations. In the following multimodal representation fusion phase, the use of Hadamard computation effectively reduces the number of trainable parameters since pure concatenation of all six vectors adds three times more parameters to the final prediction layer. In addition, other components, except for the pretrained models, in our architecture have a few layers, ensuring a reasonable computational load and a low-computational task.

### 4.4. Result of Different Modalities and Their Combinations

Similarly, in order to explore the importance of different modalities in multimodal tasks, we used speech, text, and visual features as independent inputs and conducted single-modal and bimodal experiments in which any two modalities are fused with each other, and we obtained the comparative experimental results, as shown in Tables 7 and 8.

Among them, *V* represents vision emotion analysis, *S* represents speech emotion analysis, *T* represents text emotion analysis, *V* + *S* represents speech and vision bimodal emotion analysis, *V* + *T* represents text and vision bimodal emotion analysis, *T* + *S* represents speech and text bimodal emotion analysis, and *V* + *S* + *T* represents three modal fusion emotion analysis.

From the analysis of the experimental results in Table 8, in unimodal, it can be seen that *T* shows higher performance for most emotional classes (e.g., happiness, anger, and neutral) than *S* and *V*. In the case of bimodal, *V* + *T* has the best performance, especially for sadness, anger, and neutral, which has a dominant effect on emotion recognition. Our trimodal CMC-HF model shows the highest performance for happiness, anger, and neutral by fusion of *V* + *T* + *S*. This experiment shows that under the condition of using cascade encoders, increasing the number of modalities can effectively increase accuracy, which shows that the interaction of information between different modalities is very effective and necessary for emotion recognition.

Table 9 shows the results for the CMU-MOSI dataset. Compared with other unimodal or bimodal models, our trimodal CMC-HF model has the best performance for the Acc (2 class-h), *F*1 score, MAE, and Corr metrics. The *T* + *V* model has higher Acc (7 class h), which improved by 0.008 over the trimodal CMC-HF model, which is due to the introduction of speech data causing some information redundancy.

### 4.5. Ablation Experiments

#### 4.5.1. Text Data Encoder Ablation Experiment

We studied the effectiveness of the EFC encoder model for the IEMOCAP dataset, and there are two comparison models, one is to replace the BERT-wwm model with the traditional BERT pre-training model in the text data encoding layer, and the other is to remove the CNN to form the BERT-wwm module, while keeping the other modules and parameters of the system model unchanged, and and using these two methods compared with the EFC-Encoder. From the experimental results shown in Table 10, it can be seen that the EFC encoder has the excellent performance of emotion recognition for the four classes, and the BERT-wwm model has a good performance. The improved BERT-wwm model enables the model to capture more emotional features and improve the performance of emotion recognition. And from the experimental results of BERT-wwm and EFC-Encoder, we can draw the following conclusions: The introduction of a CNN module can capture local features in text and effectively pay attention to the emotional information ignored in the BERT-wwm module. Therefore, final accuracy has improved.

#### 4.5.2. Speech Data Encoder Ablation Experiment

In order to study the effectiveness of the CSA encoder model, we conducted the comparative experiment with the transformer encoder and VGGish model, which are obtained by removing the other module in the CSA encoder model. In these experiments, the other parameters of the emotion recognition model are kept unchanged, and the experimental results obtained are shown in Table 11; we can observe that the CSA encoder model improves the other two models of the four classes by 7%∼10% in terms of accuracy. It can be seen that the CSA encoder model, effectively cascading the VGGish model and transformer encoder, has the best experimental results for four classes. On the one hand, compared with the transformer encoder model, which only uses the speech data in the IEMOCAP dataset to train the model, the introduction of the VGGish pretraining model, with a huge amount of emotional data as the basis for pretraining, can extract more expressive speech features and more emotional information and improve the generalization ability of the model.

On the other hand, in the VGGish network, the superior transformer encoder can effectively solve the problem of insufficient semantic extraction due to distance information and fully understand the contextual relationship between words, in which the multihead attention mechanism pays attention to different emotional characteristics, extracts richer semantic information, and improves the computing speed. The CSA encoder model effectively compensates for the shortcomings caused by the use of only these two single networks as a speech encoder, effectively captures the information about speech emotions, and improves the accuracy of final emotion recognition.

#### 4.5.3. Visual Data Encoder Ablation Experiment

We remove VGG16-LSTM and the Dlib library from the PC encoder separately. First, we remove VGG16-LSTM and only retain the 68 key point feature information and distance information obtained from the Dlib library as the final visual emotion feature. The other operation only uses VGG16-LSTM. The other parameters of these two models are kept unchanged. From the results in Table 12, we find that the PC encoder has the best performance; compared with that of the Dlib library or the VGG16-LSTM model, the four classes in the IEMOCAP dataset have higher accuracy. The VGG16-LSTM model effectively obtains the visual representation feature, and the introduction of sufficient prior data can provide a more robust effect for sentiment analysis and add more timing sequence information. Besides, by introducing the auxiliary features extracted by the parallel channel Dlib library, the noise data in the VGG16-LSTM model are effectively eliminated.

#### 4.5.4. Hierarchical Fusion Module Experiments

In order to verify the effectiveness of the hierarchical fusion module, we compare the two fusion stages and observe their respective effects on the performance of emotion classification:

1. We only implement the first-stage fusion and use CMC-HF (I, *∗*) to represent this ablation model. The specific operation is to remove the GCN fusion module in the model. That is, we do not consider the cross-modal fusion of text, speech, and vision, contextual emotional information, and different features between modalities, which are ignored. The final classification representation can be expressed as *U*=[*U*_*t*_, *U*_*a*_, *U*_*v*_].
2. We use CMC-HF (*∗*, II) to represent the second-stage fusion. That is, we remove the collaborative attention mechanism and the Hadamard dot product module in the model, ignore the interactive fusion of important emotional information features between two modalities, and only consider the different emotional information between modalities; the final representation is *U*=[GCN(*H*_*t*_), GCN(*H*_*a*_), GCN(*H*_*v*_)].

During this experiment, we keep the other experimental parameters unchanged, and the comparative experimental results are shown in Table 13; compared with that of CMC-HF (I, *∗*) and CMC-HF (*∗*, II), it can be seen that the performance of CMC-HF has been improved to a certain extent. CMC-HF (*∗*, II) does not emphasize the important emotional characteristics contained in two-by-two modalities, and its performance is worst. CMC-HF (I, *∗*) does not take into account different emotional features contained in the three modalities, the result in the contextual information of the three modes is insufficiently extracted, and the performance is better. CMC-HF provides sufficient interaction and fusion of important and differential affective features between modalities, and the performance is best. This experiment illustrates that it is necessary to pay attention to the important and different emotional features between the modalities in a hierarchical fusion way and that it is also essential to explore the contextual information between different modalities, which verifies the effectiveness of the hierarchical fusion strategy proposed in this paper.

## 5. Conclusions

In this paper, we propose a cascaded multichannel hierarchical fusion method for multimodal emotion recognition. First, different cascade data encoders are established for text, speech, and visual modalities for feature extraction. Specifically, the CSA encoder is designed to extract deep speech emotion features, which improves the speed of parallelized computing and further enriches semantic information; the EFC encoder is designed to encode textual emotion information with a great emphasis on global and local contributing textual features, solving the sparsity of key features; the PC encoder is designed for visual feature extraction, which effectively solves the problems of low robustness and less temporal information compared to traditional extraction methods. Second, we design a hierarchical fusion approach; in the first fusion stage, we use a collaborative attention mechanism to pay attention to contributing emotional feature intramodalities, realize the long-distance information interaction between multimodal sequences with different time steps, reduce the cost of alignment operation, and solve the long-term dependency problem in the traditional interaction network. In the second fusion stage, GCN is utilized to fuse multimodal context information feature intermodalities obtained by the Hadamard dot product, reduce the loss of emotional information, and achieve full interaction between multimodal intermodalities. Compared to other recent approaches in the literature, the proposed method achieved competitive results for two important and challenging benchmark datasets IEMOCAP and CMU-MOSI. Although the proposed method has made promising progress in performance improvement, there is a need to explore such models to extract features and ways to design fusion approaches and interaction models that can learn about joint representation between more and more modalities in future research.

## Figures and Tables

**Table 1:** Sample of the BERT-wwm model.

Description	Samples
Original text	The weather is somewhat terrible today, I'm in a completely bad mood
Input text	The weather is ##some ##what terrible today, I'm in ##complete ##ly a bad mood
BERT text	The weather is [Mask] ##what terrible today, I'm in [Mask] ##ly a [Mask] mood
BERT-wwm text	The weather is [Mask] [Mask] terrible today, I'm in [Mask] [Mask] a [Mask] mood

**Table 2:** IEMOCAP dataset allocation.

	Session 1	Session 2	Session 3	Session 4	Session 5
Anger	229	137	240	327	170
Happiness	278	327	286	303	442
Sadness	184	197	305	143	245
Neutral	384	362	320	258	384
Total	4290				1241
Percentage	77.7%				22.3%

**Table 3:** Experimental platform.

Experimental environment	Specific information
Operating system	Windows 10
GPU version	GTX 2080Ti
Development language	Python 3.6
Development platform	PyTorch
Development tools	PyCharm

**Table 4:** Comparative experiment details.

Model	Speech	Text	Vision	Fusion
MFN [43]	LSTM	LSTM	LSTM	Feature fusion
MCTN [32]	CNN	CNN	CNN	Concatenation
RAVEN [33]	LSTM	LSTM	LSTM	Feature fusion
HFusion [34]	openSMILE	Word2vec + CNN	3D CNN	Hierarchical fusion
MulT [35]	Conv 1D	Conv 1D	Conv 1D	Feature fusion
SSE-FT [40]	Wav2vec	Roberta	FabNet	Hierarchical fusion
CMC-HF(ours)	CSA encoder	EFC encoder	PC encoder	Hierarchical fusion

**Table 5:** Comparison between CMC-HF and other state-of-the-art methods for the IEMOCAP dataset.

Model	Happiness		Sadness		Anger		Neutral	
	Acc	*F*1	Acc	*F*1	Acc	*F*1	Acc	*F*1
MFN	86.5	84.0	83.5	82.1	85.0	83.7	69.6	69.2
MCTN	80.5	77.5	72.0	71.4	64.9	65.6	49.4	49.3
RAVEN	77.0	76.8	67.6	65.6	65.0	64.1	62.0	59.5
HFusion	74.3	81.4	75.6	77.0	79.6	77.6	78.4	71.2
MulT	84.8	81.9	77.7	74.4	73.9	70.2	62.5	59.7
SSE-FT	86.5	85.7	86.7	86.2	89.4	89.0	76.0	75.9
CMC-HF (ours)	**88.7**	**86.2**	**89.6**	**89.1**	**91.2**	**90.8**	**79.0**	**77.3**

The values in bold represent the best values of the attributes in this column. The larger the value of Acc and F1, the better the corresponding model.

**Table 6:** Comparison between CMC-HF and other state-of-the-art methods for the CMU-MOSI dataset.

Model	Acc (7 class h)	Acc (2 class h)	*F*1 score	MAE	Corr
MFN	34.1	77.4	77.3	0.965	0.632
MCTN	35.6	79.3	79.1	0.909	0.676
RAVEN	33.2	78.0	76.6	0.915	0.691
HFusion	35.3	77.9	79.8	0.890	0.703
MulT	39.1	81.1	81.0	0.889	0.686
SSE-FT	**55.7**	87.3	87	**0.529**	0.792
CMC-HF (ours)	**58.2**	**88.2**	**87.3**	0.581	**0.798**

The bold values represent the best values of the attributes in this column. The larger the values of Acc (7 class-h), Acc (2 class-h), F1 score, and Corr, the better the corresponding models. The smaller the value of MAE, the better the corresponding model effect.

**Table 7:** The comparison of model complexity.

Models	Number of parameters	FLOPs	Inference time (ms)
HFusion [34]	224,383,645	**253,961,225**	98.4
SSE-FT [40]	526,980,770	591,236,844	132.9
CMC-HF (ours)	**132,491,017**	261,322,124	**63.2**

The bold values represent the best values of the attributes in this column. The smaller the value of number of parameters, FLOPs, and conference time (ms), the better the effect of their corresponding models.

**Table 8:** Comparison of the experimental results for single-modal, bimodal, and multimodal emotion recognition for the IEMOCAP dataset.

Task	Happiness		Sadness		Anger		Neutral	
	Acc	*F*1	Acc	*F*1	Acc	*F*1	Acc	*F*1
Unimodal								
*V*	0.834	0.826	0.826	0.802	0.892	0.874	0.694	0.683
*S*	0.802	0.798	0.849	0.826	0.893	0.890	0.710	0.698
*T*	0.863	0.857	0.846	0.836	0.908	0.896	0.776	0.764
Bimodal								
*V* + *S*	0.802	0.794	0.878	0.869	0.906	0.880	0.725	0.718
*V* + *T*	0.849	0.836	0.865	0.862	0.910	0.901	**0.823**	**0.794**
*T* + *S*	0.856	**0.854**	0.863	0.854	0.907	0.894	0.793	0.768
Trimodal (CMC-HF)								
*V* + *S* + *T*	**0.887**	0.862	**0.896**	**0.891**	**0.912**	**0.908**	0.790	0.773

**Table 9:** Comparison of the experimental results for singlemodal, bimodal, and multimodal emotion recognition for the CMU-MOSI dataset.

Task	Acc (7 class h)	Acc (2 class h)	*F*1 score	MAE	Corr
Unimodal					
*V*	0.324	0.713	0.694	0.862	0.473
*S*	0.298	0.684	0.651	0.913	0.246
*T*	0.365	0.832	0.813	0.745	0.764
Bimodal					
*V* + *S*	0.13	0.736	0.725	1.34	0.236
*V* + *T*	**0.551**	0.869	0.856	0.738	0.824
*T* + *S*	0.436	0.834	0.816	0.917	0.794
Trimodal (CMC-HF)					
*V* + *S* + *T*	0.543	**0.882**	**0.873**	**0.581**	**0.798**

The bold values represent the best values of the attributes in this column. The larger the values of Acc (7 class-h), Acc (2 class-h), F1 score, and Corr, the better the corresponding models. The smaller the value of MAE, the better the corresponding model effect.

**Table 10:** Comparison of the experimental results in the text data encoder for the IEMOCAP dataset.

Model	Happiness		Sadness		Anger		Neutral	
	Acc	*F*1	Acc	*F*1	Acc	*F*1	Acc	*F*1
BERT	80.2	79.6	82.4	82.1	81.3	79.6	69.4	68.7
BERT-wwm	83.4	82.5	83.6	82.7	80.8	79.4	71.4	70.5
EFC encoder	88.7	86.2	89.6	89.1	91.2	90.8	79.0	77.3

**Table 11:** Comparison of the experimental results in the speech data encoder for the IEMOCAP dataset.

Model	Happiness		Sadness		Anger			Neutral	
	Acc	*F*1	Acc	*F*1		Acc	*F*1	Acc	*F*1
VGGish	79.1	79.6	80.9	79.5		81.6	79.6	71.3	69.7
Transformer encoder	84.1	83.6	86.8	86.4		87.9	86.7	74.8	73.5
CSA encoder	88.7	86.2	89.6	89.1		91.2	90.8	79.0	77.3

**Table 12:** Comparison of the experimental results in the visual data encoder for the IEMOCAP dataset.

Model	Happiness		Sadness		Anger		Neutral	
	Acc	*F*1	Acc	*F*1	Acc	*F*1	Acc	*F*1
Dlib	74.3	73.3	72.9	71.4	79.8	78.2	62.4	60.3
VGG16-LSTM	80.3	79.6	82.4	81.9	81.4	80.5	69.6	68.4
PC encoder	88.7	86.2	89.6	89.1	91.2	90.8	79.0	77.3

**Table 13:** Comparison of the experimental results for the hierarchical fusion module.

Model	Happiness		Sadness		Anger		Neutral	
	Acc	*F*1	Acc	*F*1	Acc	*F*1	Acc	*F*1
CMC-HF (*∗*, II)	76.3	75.2	78.9	78.3	80.4	79.6	69.4	68.7
CMC-HF (I, *∗*)	79.8	78.6	80.9	80.2	82.6	81.4	71.4	70.5
CMC-HF	88.7	86.2	89.6	89.1	91.2	90.8	79.0	77.3

## Data Availability

The IEMOCAP dataset used to support the findings of this study may be released upon application to the SAIL Lab at the University of Southern California which can be contacted at anfengxu@usc.edu. The CMU-MOSI dataset used to support the findings of this study has been deposited in the https://github.com/A2Zadeh/CMU-MultimodalDataSDK.
